# High Preventive Effect of G2-S16 Anionic Carbosilane Dendrimer against Sexually Transmitted HSV-2 Infection

**DOI:** 10.3390/molecules25132965

**Published:** 2020-06-28

**Authors:** Ignacio Rodriguez-Izquierdo, Samanta Gasco, Maria Angeles Muñoz-Fernández

**Affiliations:** 1 Immunology Section, Laboratorio InmunoBiología Molecular, Hospital General Universitario Gregorio Marañón (HGUGM), Instituto Investigación Sanitaria Gregorio Marañón (IiSGM), Spanish HIV HGM BioBank, C/Dr. Esquerdo 46, 28007 Madrid, Spain; igna.iri.93@gmail.com (I.R.-I.); samantagasco@gmail.com (S.G.); 2 Plataforma de Laboratorio, Hospital General Universitario Gregorio Marañón, 28007 Madrid, Spain; 3 Networking Research Center on Bioengineering, Biomaterials and Nanomedicine (CIBER-BBN), 28007 Madrid, Spain

**Keywords:** in vitro and in vivo studies, G2-S16 dendrimer, HSV-2, microbicide

## Abstract

Anionic carbosilane dendrimers such as G2-S16 are very effective in preventing HSV-2 infection both in vitro and in vivo. We present the main achievements obtained for the G2-S16 dendrimer in vivo, especially related to its efficacy against HSV-2 infection. Moreover, we discuss the mechanisms by which the G2-S16 dendrimer applied vaginally as a topical microbicide has been demonstrated to be safe and harmless for the vaginal microbiome balance, as both conditions present an essential step that has to be overcome during microbicide development. This review points to the marked protective effect of the G2-S16 dendrimer against sexually transmitted HSV-2 infection, supporting its role as a possible microbicide against HSV-2 infection.

## 1. Introduction

Dendritic structures, such as dendrimers, were officially introduced almost 40 years ago as a new class of polymers at The Winter Polymer Gordon Conference by chemist Donald Andrew Tomalia [1]. They are included in a wider nanotechnological field comprising lineal polymers, branched or hyperbranched, and cross-linked structures. These architectural classes are subsequently divided into dendritic subclasses, including dendrimers and dendrons [2,3]. Dendrimers are one of the most studied and applied molecules. They are nanosized (1 to 20 nm), star-shaped, and highly branched structures. Their general structure includes the central core, the repetitive branched units, and the peripheral groups on the dendrimer surface [4,5,6]. The density of the functional groups in the periphery increases exponentially with each generation, while the diameter grows around 1nm/generation [7]. The morphology of the dendrimer is determined by the core and the type of the functional groups, its internal properties are controlled by the interior structure, and the number of the dendrimer is defined by the number of repetitive layers group [8,9]. Finally, the generation number of the dendrimer is defined by the number of repetitive layers from the core to the surface. The most widely-used nomenclature names the central core as G0, and the successive branching units corresponding to each next generation as G1, G2, G3, and so on. This classification is not rigid, though, as the generation could be defined by the number of layers, the repetitive units, or other similar criteria. Hence, when a dendrimer is presented, this classification must be clearly defined. The flexibility and shape of the dendrimer are also related to the generation size, so that the lower dendrimer generations are generally less spherical and usually adopt a planar structure, while the dendrimers belonging to higher generations are usually globular and compact, and this characteristic is also related to the viscosity level of the dendrimer [10,11]. On the other hand, as aforementioned, the functional groups define the physicochemical properties of the molecule. The interactions between the dendrimer and the cellular receptors or other molecules are defined by the presence and the density of the chains in its periphery. Moreover, the type and the nature of the functional group also defines the solubility and the reactivity of the dendrimer.

There are two main synthesis methods to generate dendrimers: the convergent or the divergent method. Although most of the dendrimers are synthesized using the convergent method, i.e., the growth of the branched structure starts from the dendrimer core, the divergent method (starting from the branches into the dendrimer core) is very interesting due to the possibility of making polyvalent modifications to the dendrimer [12]. The divergent method involves two main steps: the serial addition of the repeated branching units to the core and the activation of the functional end groups. It has some advantages, such as the ability to modify the functional groups at the most external layer, thus adapting the compound to specific functional needs. On the other hand, the convergent method also comprises two main steps: the generation of several individual branched units attached to a core (dendrons), and the junction of those individual dendrons to a central core to generate a complete dendrimer [13]. In the convergent method, the weight of the dendrimer and the integrity of the functional groups allow for a tighter control. This is an important advantage, as it provides protection for the active sites along the several necessary reactions of dendrimer synthesis, and this is one of the main goals and challenges during the scaling-up process. There are other technologies to synthesize dendrimers, such as the double exponential growth, the “click” and “lego” chemistry or the hyper-cores, and branched monomers growth [10,11]. However, it is not clear enough which is the best cost-effective method, and more studies related to dendrimer synthesis must be conducted to clarify this issue.

Dendrimers have been widely used in several fields, such as nanomedicine, technology, and industry, among others. Therefore, a perfect and well-defined characterization of each dendrimer is crucial, as dendrimer properties will define their specific use and application [14]. In this sense, it is crucial for the researcher to use the needed analytic tools to determine dendrimers properties such as size, purity, shapes, or chemical structure, taking in mind the techniques limitations. Nowadays, there exists a great diversity of dendrimers, based on their properties and chemical structure: dendrimers based on a carbon oxygen (such as polyester glycodendrimers), dendrimers based on silicon (silane, carbosilane, carbosiloxane, siloxane) such as G2-S16 dendrimer, triazine dendrimers, polyamidoamine (PAMAM) dendrimers, metallic dendrimers (metallodendrimers), PLL (polylysine) dendrimers, chiral dendrimers, peptide dendrimers, polyamidoamine/organosilicon (PAMAMOS) dendrimers, porphyrin-based dendrimers, dendrimers based on phosphorus-based dendrimers, or polypropylene-imine (PPI) dendrimers [10,14,15]. The wide arrays of dendrimers types place these nanoparticles as potential candidates for biomedical applications, but it is crucial to consider that the surface functional groups of the dendrimers have an important effect on its distribution, metabolism, absorption, and elimination within the organism [6], so that some types would be more adequate than others for their use as therapeutic molecules. Cationic dendrimers interact with the negative charges of the lipid bilayer, decreasing its integrity and/or blocking membrane cellular receptors, a fact that could finally cause its destabilization and the subsequent cellular death [16], thus not being useful for biomedical use. Anionic dendrimers, on the contrary, are mostly compatible with biomedical applications, as they possess positive charges and respect the cell membrane integrity. Thus, one of the potential applications of anionic carbosilane dendrimers is in the form of microbicides [17,18,19].

The potential applications of anionic dendrimers are very broad, as they could be used as antiviral and antibacterial agents [17,20,21,22,23], as carriers of chemical drugs and peptides [24,25,26,27,28], as gene silencers [29], and as potential therapeutic agents for diverse pathological conditions, including neurodegenerative diseases (such as Alzheimer´s disease) [30], cancer [31,32,33], chronic inflammatory diseases [34,35] and infectious diseases [36], among others.

Currently, the development of new microbicides revolves heavily around the use of nanotechnology, as it provides a set of state-of-the-art tools to generate effective and safe microbicides against a wide array of infections [37,38]. Particularly, as aforementioned, dendrimers have been demonstrated to present a broad range of applications, mainly in nanomedicine [9,39], and they have a high relevance as antiviral agents against a wide array of viruses [40,41,42,43,44,45]. One of the main problems related to the microbicides is that, despite the great results obtained in vitro and in vivo, the last steps of the clinical trials usually fail, so the preclinical assessment must be more exhaustive. Evaluating the activity of microbicide candidates mimicking as much as possible the conditions present at the early stages of HSV-2 infection in more physiological conditions is crucial. The assessment of a new topical microbicide should study the impact on sexual fluids, on the mucosal immune response, its effect in presence of sexual physical trauma and potential epithelial injuries derived, as well as, assessing inflammatory consequences of its application [46]. Hence, in this review, we present a detailed description of the potential usefulness of the G2-S16 polyanionic carbosilane dendrimer in the treatment of HSV-2 infection.

## 2. HSV-2 Infection Idiosyncrasy and HSV-2 Morphology and Structure

Herpes Simplex Virus are DNA viruses belonging to the *Alphaherpes viridae* subfamily, within the Herpesviridae family, where herpes type 1 and 2 (HSV-1 and HSV-2) are the most predominant ones [47]. Infection by these viruses involves the most prevalent sexual transmission infections (STIs) in industrialized and developing countries [48,49]. Approximately 19 million new HSV-2 infections occur every year, and 417 million people have genital herpes caused by HSV-2 worldwide [50]. Given that asymptomatic HSV-2 reactivation and shedding are common, around 90% of the individuals worldwide are not aware of being infected [48,49,51,52]. The majority of HSV-2 transmissions occur during the asymptomatic stage [53], although this virus is associated with considerable morbidity and mortality [54]. The main symptoms caused by HSV-2 infection are pain, dysuria, urethral painful, inguinal lymphadenopathy, and vaginal discharge [55]. Short-term treatment or sporadic therapy with antiviral agents such as acyclovir, famciclovir, and valacyclovir drugs reduce symptoms and the frequency of recurrences and shortens the duration of the tissue injury [56,57,58,59,60,61,62]. Patients who received maintained suppressive treatment had fewer episodic recurrences, although sustained treatment with these drugs should be avoided, as it can induce the emergence of drug-resistant HSV-2 [63,64,65,66]. Currently, there is no cure or effective vaccine for HSV-2 infection, and the treatment is focused on reducing the clinical symptoms using current antiviral drugs. However, HSV-2 latent infection cannot be eliminated [50,63,64,67,68,69,70]. The main HSV-2 contagion route is heterosexual contact. In developing countries, women are at an even higher risk of acquiring HSV-2 infection, as they are not able to discuss fidelity issues, to leave risky relationships, or to negotiate the use of a condom. Regarding this, the development of a 2 vaginal microbicide effective against HSV-2 and other STIs would greatly aid in the prevention of these STIs.

**Figure 1:**
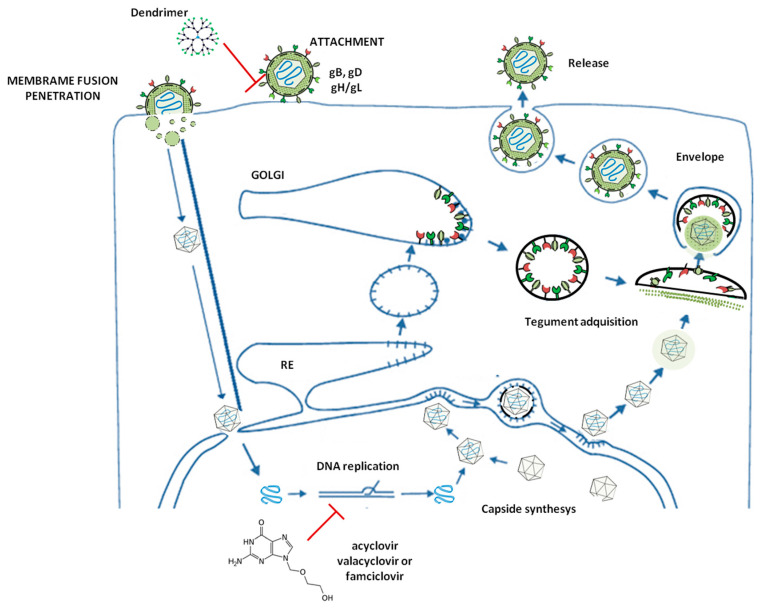
The infective cycle of HSV-2. Schematic representation of the HSV-2 infective life cycle and the possible steps where antivirals can perform their action.

With regard to HSV-2, the virions present a complex composition, consisting of a core containing a double-stranded DNA genome, composed by 155kbp. This genome contains two major regions, large and small (UL and US), as well as two inverted repeat elements (TRLs) [71,72]. The genome is enclosed by a capsid with an icosapentahedral structure. The tegument, which is an amorphous protein pool, coats the icosapentahedral capsid and, finally, the whole virion is encapsulated by a lipid bilayer with the necessary glycoproteins for the viral entry [73]. Related to the complex genome, 74 ORFs have been identified to date. The UL and US regions contain 56 and 12 viral genes, respectively, all of them transcribed by the RNA polymerase II of the infected cells [74]. The RTLs make HSV-2 highly variable, given that they make it possible to invert the UL or the US, hence generating a variety of isoforms [75]. HSV-2 infection is mediated by the interaction between the viral envelope glycoproteins (gD, gB, and the heterodimer gH/gL) and the cellular receptors of the plasma membrane, such as heparin sulfate proteoglycans (HSPG) [76,77,78]. Once these primary interactions occur between gB and HSPG, the gD interacts specifically with cellular receptors such as HVEM (herpesvirus entry mediator) or nectin-1. This union unchains the fusion membrane process mediated by gH/gL heterodimer [79,80] (Figure 1).

Once the viral and the host cell membranes are fused, the capside is transported to the nucleus by the cellular microtubules, the viral genome enters the nucleus through the nuclear pores, and the early viral genes being to get transcribed rapidly [52] (Figure 1). These genes are capable of inhibiting several cellular antiviral mechanisms [53]. Afterward, the early and late genes have been synthesized, and the HSV-2 viral capsid fuses with the integument and the surface glycoproteins of the virion. Finally, the new virions get exported into the extracellular medium (Figure 1).

There are several steps in this viral cycle where the antivirals can act (Figure 1). For example, the acyclovir, famciclovir, and valacyclovir drugs inhibit the translation process, while dendrimers act in the first steps of the infection, such as viral entry, by interacting with the HSPG [18].

## 3. G2-S16 Anionic Carbosilane Dendrimer

**Figure 2:**
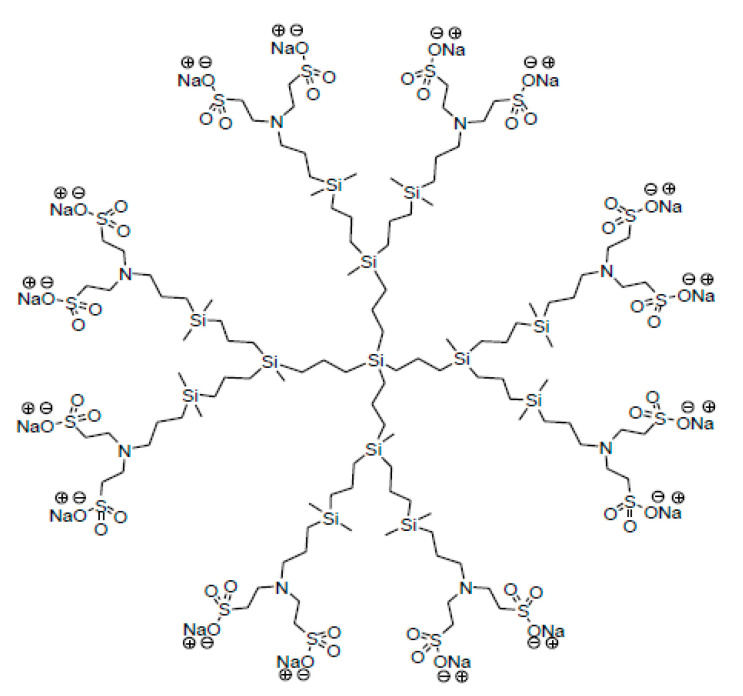
Schematic representation of the G2-S16 anionic carbosilane dendrimer.

As aforementioned, dendrimers are nano-sized molecules, with a tree-like structure usually generated by the addition of branching units from a central core [2,4,5,6,8,50,81]. The possibility of selecting different cores, as well as the number and type of branching units and the peripheral functional groups, provides dendrimers with a wide array of reactive sites and properties [9]. Even though every year thousands of nanocompounds and dendrimers are published, only the G2-S16 dendrimer will be described in this work [14]. The stable and water-soluble G2-S16 anionic carbosilane dendrimer (Figure 2) was synthesized according to the methods reported by the Dendrimers for Biomedical Applications research group (BioInDen) of the University of Alcala (Madrid, Spain) [21,23,82]. The G2-S16 dendrimer is a second-generation dendrimer presenting 16 sulfonate groups in its periphery. The generation is defined as the number of repeated layers of branching units forming the dendrimer, as mentioned above. The G2-S16 dendrimer presents a silicon core and has a molecular weight of 3,712.78 g mol^−1^ and its molecular formula is C_112_H_244_N_8_Na_16_O_48_S_16_Si_13_. The final product was obtained as a white solid from G_2_Si(NH_2_)_8_ (0.50 g, 0.31 mmol) and C_2_H_3_SO_3_Na (5.20 mmol).

It is important to note that, before working with the G2-S16 dendrimer, its chemical composition, shape, size, morphology, homogeneity and purity or monodispersity among other properties must be clearly defined, just as for any type of dendrimer [23].

## 4. G2-S16 Anionic Carbosilane Dendrimer as an In Vivo Vaginal Microbicide

**Figure 3:**
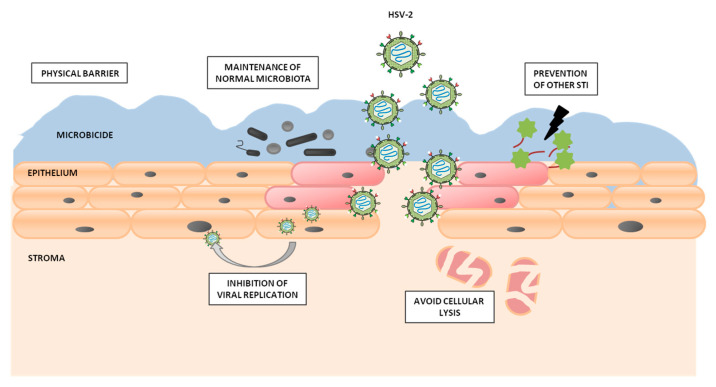
Schematic representation of the main goals in microbicide development. Microbicides must act as a lubricating layer that creates a physical barrier against infection with HSV-2, while also maintaining the vaginal defenses and inactivating the virus, even if it is able to cross the epithelial barrier.

Some characteristics of the ideal vaginal microbicide (Figure 3) include displaying a significant activity against HSV-2 and other STI etiologic agents, working over a broad pH range, retaining its activity over time, even in the presence of sexual fluids such as semen, and not disrupting the vaginal flora or the structural integrity of the epithelium. Other important factors such as odor, color, or taste are very important too. In addition, topical microbicides must be stable at relatively high temperatures and easy to handle, should have a long shelf-life, and a reasonable price to make them accessible, among other desired features [67,83].

As a potential microbicide, the G2-S16 dendrimer has been formulated in a water-based gel format because gels are optimal formulations to ensure that the microbicide begins to act quickly and stays in close contact with the target tissue. The vehicle used as a carrier is a hydroxyethyl-cellulose gel [84] and the active pharmaceutical ingredient is the G2-S16 dendrimer. Therefore, 3% weight/volume (w/v) of the G2-S16 dendrimer is mixed in 1% (w/v) of HEC, which is a compound showing big biocompatibility with the female normal human vagina [18,85,86]. This fact is of vital importance, given that surfactants such as Savvy gel^®^ or nonoxynol-9, which have been widely used in the topical microbicide design, have been described to produce vaginal lesions in vivo, thus increasing the risk of sexual infections and diminishing the effectiveness of the selected active compound [87,88,89].

One fact that has critical importance and must be taken into account in the development of new vaginal microbicides is the potential appearance of interactions between the dendrimer and the vaginal environment. This is crucial, given that the activity of vaginal microbicides can be altered by the physiological characteristics of the vagina and, inversely and importantly, the dendrimer could potentially affect the integrity and homeostatic balance of the vaginal mucosa. With regard to the G2-S16 dendrimer, our group showed the safety of the G2-S16 dendrimer gel used as a topical treatment on the female vaginal mucosa. This study was performed using the CD1 (ICR) or BALB/c mouse model. Different concentrations of G2-S16 dendrimer were selected, according to previous studies found in the scientific literature that used a comparable compound, the SPL7013 dendrimer [90]. Two dendrimer applications were administered in a 24-h manner for 7 consecutive days at different doses in these CD1(ICR) or BALB/c female mice models. Histopathological examination was performed after administering doses of 1.5%, 3%, and 4.5% G2-S16 dendrimer gel, respectively, to CD1 (ICR) or BALB/c female mice and no irritation, inflammation, lesions, or damage in the vaginal mucosa were found [91]. In addition, the vaginal G2-S16 dendrimer bio-distribution study performed in female BALB/c using a 3% G2-S16 dendrimer concentration determined that it did not cross the epithelium barrier.

The concentration reached by the G2-S16 dendrimer is higher than that for drugs tested as vaginal microbicides and which are in the clinical trial phase [92,93], therefore positioning the G2-S16 dendrimer as a very promising candidate, given that the higher the biocompatible concentration a dendrimer achieves, the bigger the effect against viral infections.

In order to perform further safety studies of the G2-S16 dendrimer, we first chose mice [40,87,93], then female rabbits as the preferred animal model due to the high degree of histopathological similarity between the rabbit and the human vagina. We showed that the G2-S16 dendrimer is well tolerated and does not show any severe adverse events related to vaginal irritation in New Zealand white rabbits at 1 µM and 10 µM doses. No vaginal lesions, irritation or inflammation were detected in this animal model after using repeated doses of the G2-S16 dendrimer [86].

**Figure 4:**
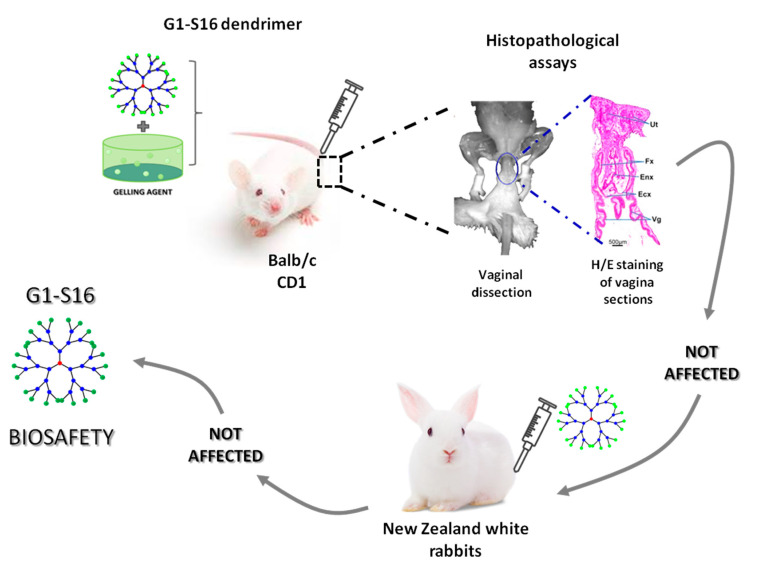
Summary of the main in vivo results obtained from the G2-S16 anionic carbosilane dendrimer study. This G2-S16 dendrimer has been demonstrated to be safe in several in vivo models such as BALB/c or CD1 female mouse, as well as in New Zealand white rabbits at different concentrations [18,85,91]. H/E: hematoxilin/eosin.

After confirming the biosafety and tolerability of the G2-S16 dendrimer as a topical vaginal microbicide in two different in vivo mice and rabbit animal models (Figure 4), we also proposed that the mechanism of action against HSV-2 infection is firstly based on the generation of a gel barrier coat on the vagina epithelium. Once the gel is applied, the G2-S16 dendrimer generates a physical barrier to prevent the infection, avoiding the dissemination from the local mucosa to the lymph nodes and acting against the HSV-2 that crossed the epithelial barrier. The fact that this microbicide is applied as a gel makes it very interesting, due to other microbicides tested being applied as films, or time-delivery systems reflecting significant variations in the rates of drug concentrations and acceptability [94,95,96]. These variations could generate resistant mutations since the selective pressure of the drug fluctuates along the exposure time. In the case of the G2-S16 dendrimer, women could apply the gel only when they presage a sexual transmission risk, being able to reach an optimal concentration of the microbicide in all selected times. The G2-S16 dendrimer was also assessed for resistance mutations, and it was demonstrated that this dendrimer does not generate resistance in cell cultures [97].

## 5. Prevention of Vaginal HSV-2 Infection in Presence of G2-S16 Anionic Carbosilane Dendrimer in Female Mice

**Figure 5:**
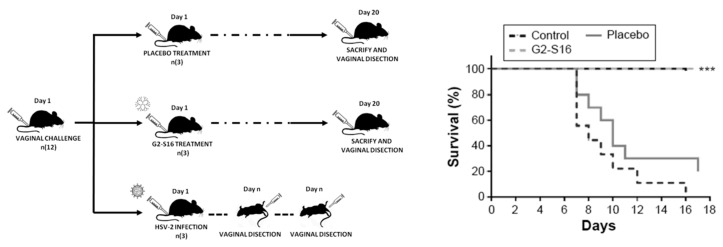
Summary of the experiment performed to check the G2-S16 dendrimer effectivity against HSV-2 infection. The G2-S16 dendrimer prevented HSV-2 vaginal infection even when using high viral doses. The Kaplan-Meier graph shows the survival rates for every group. Dendrimer-based gels containing 3% G2-S16 dendrimer were significantly more protective than the gel containing the vehicle alone (*** *p* < 0.001 when comparing treated versus placebo groups). Control group: HSV-2 infection control. Placebo group: gel vehicle only [18].

The in vivo efficacy of the G2-S16 dendrimer in the prevention of vaginal HSV-2 transmission has been demonstrated [18]. We analyzed the inhibitory activity of the G2-S16 dendrimer against HSV-2 infection in BALB/c female mice, with the objective to prove the prevention of HSV-2 transmission achieved by the use of this dendrimer [91,98,99,100]. Mice were treated vaginally with G2-S16 dendrimer and then infected with HSV-2 at a lethal dose. The results obtained demonstrated that the G2-S16 dendrimer showed a great anti-HSV-2 activity against HSV-2 clinical viral strains and proved to be able to halt HSV-2 infection in 100% of the treated female BALB/c female mice upon exposure to a lethal dose of HSV-2 (Figure 5). Most importantly, the differences found between the G2-S16-treated group, the control, and the placebo groups were very significant. As aforementioned, not only did the G2-S16 treatment halt the infection, but also no signs of HSV-2 could be traced in any of the BALB/c female mice. This G2-S16 dendrimer provides effective protection against HSV-2 infection, most likely due to its ability to bind the host cells and to create a barrier against HSV-2 entry, rather than being due to a neutralization of the virus itself. Summing up, the G2-S16 dendrimer was shown to provide complete protection against vaginal HSV-2 infection in BALB/c female mice.

Compared to other clinical trials, such as the VOICE trial, which involved Tenofovir gel for the prevention of HSV-2 infection [101], the in vivo results obtained with the G2-S16 dendrimer highlighted its potential to reach clinical trials and provide results that could equal or even exceed the results obtained in clinical trials testing other dendrimers.

The G2-S16 dendrimer can be potentially considered as an ideal topical microbicide to be applied in the vaginal mucosa as it has been proven to have a very substantial effect while still being noncytotoxic at the effective therapeutic concentrations easily achievable in BALB/c female mice [18]. Moreover, our group also studied the anti-HSV-2 activity of the G2-S16 dendrimer at different pHs values and proved that the vaginal pH exerted no significant influence on the antiherpetic activity of this G2-S16 dendrimer.

There are other clinical trials related to the inhibition of STI using vaginal rings with Dapivirin (DPV) as the active compound [102,103]. A ring containing DPV was in phase 3 of the clinical trial [104], and the results showed a 37% protection, increasing up to 56% in women over 21 years of age. Our group has demonstrated that the combination of the G2-S16 dendrimer with the DPV improves the anti-HSV-2 profile of the DPV and achieves better results in HIV-1 inhibition [105]. On the other hand, the relationship between bacterial vaginosis (BV) and the possible efficacy of microbicides has been a focus of attention. A recent study revealed that the lack of effectiveness in TFV gel microbicide CAPRISA 004 clinical trial was due to the present BV and to the drug depletion via bacterial metabolism [106,107]. These results support the idea that G2-S16 dendrimer represents a safe and powerful candidate to step into HSV-2 treatment clinical trials (*manuscript under preparation*). Our results indicate that the G2-S16 dendrimer could be taken to human preclinical trials to assess its safety and tolerability in women.

## 6. Conclusions

Dendrimers are new nanotechnology tools that have been demonstrated to have antiviral properties against a wide gamma of viral agents. In this sense, our group has been studying the antiviral properties of carbosilane dendrimers for a decade. The antiviral properties of G2-S16 dendrimer were studied by our group and we demonstrated that this dendrimer is able to inhibit the HSV-2 infection not only in vitro but also in vivo. Moreover, it was proven that its underlying mechanism of action relies on the inhibition of HSV-2 infection. Given the promising results presented in this review, concerning, the G2-S16 dendrimer biocompatibility, maintenance of the integrity of the mucosal tissues, non-inflammatory effect, inhibition effect and combination with a synthetic method, the G2-S16 dendrimer could be a good, safe, and readily-available candidate for using as a topical vaginal microbicide against HSV-2 infection.
